# Effects of dietary fermented *Saccharomyces cerevisiae* extract (Hilyses) supplementation on growth, hematology, immunity, antioxidants, and intestinal health in Nile tilapia

**DOI:** 10.1038/s41598-024-62589-9

**Published:** 2024-05-31

**Authors:** Asmaa S. Abd El-Naby, Amel M. El Asely, Mona N. Hussein, Abd El-Rahman A. Khattaby, Eman A. Sabry, Mohamed Abdelsalam, Fatma Samir

**Affiliations:** 1 Department of Fish Nutrition, Central Laboratory for Aquaculture Research Centre, Agriculture Research Centre, Abassa, Abu Hammad, Sharkia Egypt; 2 Department of Aquatic Animal Medicine, Faculty of Veterinary Medicine, Benha University, Benha, 13736 Egypt; 3 Department of Histology and Cytology, Faculty of Veterinary Medicine, Benha University, Benha, 13736 Egypt; 4 Department of Production and Aquaculture Systems, Central Laboratory for Aquaculture Research Centre, Agriculture Research Centre, Abassa, Abu Hammad, Sharkia Egypt; 5 Department of Aquatic Animal Medicine and Management, Faculty of Veterinary Medicine, Cairo University, Giza, 11221 Egypt

**Keywords:** Antioxidant activity, Immunity, Intestine morphology, Nile tilapia, Yeast extract, Ichthyology, Cell biology

## Abstract

This study investigated the effects of dietary supplementation with the product Hilyses on growth performance, feed utilization, nutrient composition, hematological parameters, serum biochemistry, immune function, antioxidant status, and digestive enzyme activity in juvenile Nile tilapia (*Oreochromis niloticus*, initial body weight 4.24 ± 0.01 g). The fish were fed diets supplemented with Hilyses at concentrations of 0, 1, 2, or 3 g/kg for a period of 8 weeks. The results showed that supplementation with Hilyses at levels up to 2 g/kg diet significantly improved final body weight, weight gain, specific growth rate, feed efficiency ratio, protein efficiency ratio, apparent protein utilization, and energy utilization compared to the control diet without Hilyses. Carcass crude protein content and moisture were significantly higher in Hilyses-fed groups, while crude lipid content decreased at the 3 g/kg supplementation level. Hilyses supplementation enhanced various hematological parameters, including increased red blood cell count, total leukocyte count, hemoglobin concentration, hematocrit, and mean corpuscular volume. Serum biochemistry and immune function markers like total protein, albumin, complement component C3, IgM, and IgG were significantly elevated in the 2 and 3 g/kg Hilyses groups. Antioxidant enzyme activities (catalase, glutathione peroxidase, total superoxide dismutase) were enhanced, and lipid peroxidation was reduced, in the 2 g/kg Hilyses group. Digestive enzyme activities, particularly protease and lipase, were also improved with Hilyses supplementation. Histological examination showed reduced lipid deposition in the liver and increased branching of intestinal villi at the 2 g/kg Hilyses level. Overall, these results indicated that dietary Hilyses supplementation at 2 g/kg diet optimizes growth, feed utilization, nutrient composition, hematology, immunity, antioxidant status, and digestive function in juvenile Nile tilapia.

## Introduction

Aquaculture has experienced rapid growth, emerging as the fastest-expanding global food production sector. Current projections indicate that aquatic food production will increase by 15% by 2030^1^, underscoring the need for more efficient and sustainable production methods. With escalating aquaculture output, enhancing disease control and boosting immunity through optimized nutrition has become imperative^2,3^. Extensive studies on fish nutrition highlight the crucial role of amino acids and other forms of dietary protein in bolstering the immune systems of fish^4,5^. However, fish feeds based on plant ingredients often suffer from deficiencies in digestibility and protein quality^6^. To address these nutritional deficiencies, numerous commercial feed additives have been developed as targeted supplements. One such additive that stands out is Hilyses, a unique nutraceutical formulation derived from the fermented *S. cerevisiae*, a versatile microorganism with numerous applications in aquaculture. Hilyses contains a diverse array of bioactive compounds, including free nucleotides, nucleosides, glutamine, peptides, β-glucans, and mannan oligosaccharides (MOS)^7,8^. This product is specifically tailored to stimulate digestive functions, enhance antioxidant capabilities, regulate pathogenic bacteria, and promote a healthy balance of intestinal flora^9^. The efficacy of Hilyses is attributed to the synergistic interplay of these active constituents, each of which can contribute to the overall improvement of fish health and performance.

Glutamine, for instance, is one of the most abundant free amino acids in fish plasma and tissues, and it serves multiple crucial physiological roles^10^. It is a key player in protein synthesis, an important energy source for leukocytes, and plays a vital role in controlling cytokine and nitric oxide (NO) synthesis, thus contributing significantly to the immune response in fish^5,10^. Supplementation with glutamine has also been found to have a normalizing effect on lymphocyte populations and function, as well as enhancing the structure and functionality of the intestine in juvenile carp^11,12^. Another important component of Hilyses is the antimicrobial peptides (AMPs), which are small proteins composed of amino acids that can eliminate a broad spectrum of infections^13,14^. As part of the innate immune system, AMPs play a crucial role in eliminating pathogens by disrupting bacterial membranes. Previous studies have demonstrated that applying exogenous AMPs enhances the natural immune response and antioxidant function in aquatic organisms^15,16^. The combination of these bioactive compounds, including glutamine, AMPs, β-glucans, and MOS, allows Hilyses to exert a myriad of beneficial effects on fish health, including boosting immunity, enhancing disease resistance, improving growth performance, and increasing feed efficiency^17,18^. β-Glucans are glucose polymers that can stimulate cellular and humoral immune responses, while MOS exhibit prebiotic effects by selectively promoting the growth of beneficial gut bacteria. The prebiotic properties of MOS and β-glucan, in combination with the inclusion of glutamine, a key amino acid with immunomodulatory functions, make Hilyses a unique nutraceutical blend tailored to support overall fish health and performance. The synergistic effects of these bioactive compounds allow Hilyses to exert positive impacts on various aspects of fish physiology, including digestive function, antioxidant capacity, and disease resistance^17,18^.

Tilapia is an economically important aquaculture species, but the intensive rearing conditions often make the fish prone to infections^19,20^. The hypothesis is that supplementing the diets of tilapia with Hilyses would enhance growth perform.

ance, strengthen immunity, and improve gut health. While Hilyses has been investigated in some fish species, such as rainbow trout (*Oncorhynchus mykiss*)^7,21,22^ and orange-spotted grouper^23^, limited studies have been conducted on its impacts on Nile tilapia (*Oreochromis niloticus*)^19,20^, a widely cultured and economically significant aquaculture species.

The current study aimed to assess the impacts of supplementing varying levels of Hilyses in the diets of Nile tilapia. The evaluated parameters included growth performance, feed utilization, proximate body composition, hematological and serum biochemical indices, antioxidant enzyme activities, digestive enzyme function, and histological alterations in the intestine and liver. By elucidating the optimal supplementation level of Hilyses, this study provided valuable insights into its potential application as a dietary additive to enhance the culture and productivity of Nile tilapia, a species of global importance in the aquaculture industry.

## Material and methods

### Experimental diets

A completely randomized design with four dietary treatments in triplicate was implemented to assess the effects of supplementing a commercial nutraceutical product (Hilyses, ICC Industrial Comércio Exportaçãoe Importação SA, São Paulo, Brazil) in tilapia diets. The product was purchased from (Peacock International, Egypt), it is a completely pure yeast culture, containing no genetically modified organisms (GMOs), fillers, or carriers. Hilyses is a mixture of 63 g/kg nucleotides, 235 g/kg β-glucans and 142 g/kg MOS. Hilyses was incorporated into the diets at concentrations of 1 g/kg, 2 g/kg, 3 g/kg and, control without supplementation (0 g/kg) [Concentrations were chosen based on earlier investigations^19^ and our pilot study in the laboratory]. All diets were formulated to be isoproteic (30% crude protein) and isolipidic (7% lipid). Chemical analysis (%) including crude protein, crude lipid, moisture, ash, and crude fiber were determined for the primary ingredients using standard methods^24^ and are shown in Table 1. The experimental diets were prepared by precisely weighing out each ingredient on a digital scale according to the formulation. Ingredients were thoroughly blended along with vitamin and mineral premixes, antioxidants, and binding agents to obtain a homogeneous mixture. Water was added to obtain an appropriate consistency to produce 2 mm diameter pellets using a pellet extruder. The moist pellets were dried in a forced air oven at 60 °C for 24 h. All diets were kept frozen at − 20 °C until used. Gross energy was calculated as 5.65 kcal/g protein, 9.45 kcal/g lipid, and 4.11 kcal/g carbohydrate^25^.

**Table 1:** Ingredients and analyzed composition of the basal diet (air-dry basis, g/kg).

Ingredients	Control	Hilyses^®^ (g/Kg)		
	0.0	1.0	2.0	3.0
Fish meal (HFM)	80.0	80.0	80.0	80.0
Soybean meal (SBM)	430.0	430.0	430.0	430.0
Ground corn (CNM)	200.0	200.0	200.0	200.0
Wheat bran (WB)	180.0	180.0	180.0	180.0
Cod fish oil	30.0	30.0	30.0	30.0
Vegetable oil	15.0	15.0	15.0	15.0
Vitamins and Mineral premix^1^	15.0	15.0	15.0	15.0
Di- Calcium phosphate	10.0	10.0	10.0	10.0
Starch	40.0	39.0	38.0	37.0
Hilyses®	0	1.0	2.0	3.0
Total	1000	1000	1000	1000
Chemical analysis (%)				
Dry matter	92.1	92.2	92.5	92.8
Crude protein	30.1	30.2	30.3	30.4
Crude fat	7.5	7.7	7.8	7.9
Ash	8.2	8.3	8.4	8.6
Fiber	5.0	4.9	4.9	4.8
NFE^2^	49.2	48.9	48.6	48.3
GE (MJ/Kg)^3^	16.92	16.97	16.98	17.00

^1^Vitamin and mineral mixture each 1 kg of the mixture contains 4800 IU Vitamin A, 2400 IU cholecalciferol (Vitamin D), 40 g of Vitamin E, 8 g of Vitamin K, 4.0 g of Vitamin B12, 4.0 g of Vitamin B2, 6 g Vitamin B6, 4.0 g pantothenic acid, 8.0 g nicotinic acid, 400 mg folic acid, 20 mg biotin, 200 gm choline, 4 g copper, 0.4 g iodine, 12 g iron, 22 g manganese, 22 g zinc, 0.04 g selenium, 1.2 mg folic acid; 12 mg niacin; 26 mg D-calcium pantothenate; 6 mg pyridoxine HCl; 7.2 mg ribo £ avin; 1.2 mg thiamine HCl; 3077 mg sodium chloride (NaCl, 39% Na, 61% Cl); 65 mg ferrous sulphate (FeSO4. 7H2O, 20% Fe); 89 mg manganese sulphate (MnSO4, 36% Mn); 150 mg zinc sulphate (ZnSO4. 7H2O, 40% Zn); 28 mg copper sulphate (Cu- SO4 5H2O, 25% Cu); 11 mg potassium iodide (KI, 24% K, 76% I); 1000 mg Celite AW521 (acid-washed diatomaceous earth silica). w% on dry matter (DM) basis.

^2^Nitrogen-Free Extract (calculated by difference) = 100 – (protein + lipid + ash + fiber).

^3^Gross energy was calculated using the factors as follows: protein, 23 MJ/kg; lipid, 35 MJ/kg; carbohydrates, 15 MJ/kg (Molina‐Poveda et al., 2013).

### Fish and experimental management

The feeding trial protocols complied with the guidelines for the use of fish in research according to the American Fisheries Society (AFS, 2014) and were approved by the research ethics board of Central Laboratory for Aquaculture Research (CLAR), Abassa, Egypt (Approval no. 43429).

Nile tilapia (*O. niloticus*) fingerlings were obtained from nursery ponds at CLAR, ensuring they exhibited apparent health and were free from any pathogenic microorganisms. Fish were transported to the wet laboratory facility and acclimated to laboratory conditions for 2 weeks prior to experimentation. During acclimation, fish were fed a control pellet diet to satiation twice daily. For the feeding trial, 240 healthy tilapia juveniles with a mean initial body weight of 4.24 ± 0.1 g were randomly distributed into 12 tanks (40 × 40 × 50 cm; 80L capacity) at a stocking density of 20 fish per tank, comprising three replicate tanks per dietary treatment. Each tank was continuously aerated using air stones connected to a central air compressor. Fish were hand-fed experimental diets to apparent satiation twice per day (at 9 AM and 3 PM) for 8 consecutive weeks. During the first 4 weeks, the daily feeding rate was set at 4% of the biomass in each tank, which was then reduced to 3% for the remainder of the trial. Throughout the experiment, uneaten feed and feces were siphoned out daily, and approximately 30% of the water was exchanged with freshwater to maintain water quality. The replacement water was from a reservoir tank and had been aerated continuously for > 2 days before use. Moribund or dead fish were removed daily, and mortality was recorded.

### Water quality parameters

Water quality parameters were monitored weekly in each tank. Temperature and dissolved oxygen (DO) were measured on-site using a portable meter (Jenway, London, UK). A digital pH meter (model 55, Fisher Scientific, Denver, CO, USA) was used to measure pH. Total ammonia–nitrogen (TAN) concentrations were quantified using a multi-parameter ion analyzer (HANNA Instruments, USA). The concentration of unionized ammonia (NH3) was determined using a Multi-parameter Ion Analyzer (HANNA Instruments, Rhode Island, USA). Water temperature averaged 28 ± 2 °C, DO ranged from 5.7 ± 2.32 mg/L, pH ranged from 7.9 ± 1.2, and ammonia nitrogen ranged from 0.03 ± 0.01 mg/L. All parameters were maintained within acceptable ranges for Nile tilapia aquaculture^26^**.**

### Diet and fish sample analysis

Samples of both the diet and whole fish were collected before and after the experiment to analyze their diets proximate compositions. This analysis followed^24^ methodology and included measurements of moisture, protein, lipid, fiber, and ash content. Moisture content was determined through oven drying at 105 °C until a consistent dry weight was achieved. Ash content was determined by subjecting the sample to a muffle furnace at 550 °C for 6 h. Nitrogen content was quantified using the micro-Kjeldahl method, and crude protein was calculated by multiplying the total nitrogen content by a factor of 6.25. Lipid content was assessed by a 16-h petroleum ether extraction using the Soxhlet apparatus. Crude fiber estimation followed the method outlined in Ref.^27^. Gross energy content was calculated based on the parameters specified in Ref.^25^**.**

### Growth performance and feed utilization

At the end of the feeding trial, fish were collected, counted, measured to the nearest millimeter, and weighed to the nearest gram. Growth performance and feed utilization parameters were calculated using standard formulas of Ref.^25^ (Table 2).

**Table 2:** Growth performance and feed utilization of Nile tilapia in response to dietary additives of Hilyses^®^ for 8 weeks.

Items	Hilyses® (g/ kg)			
	0	1	2	3
Initial weight (g)	4.24 ± 0.01	4.23 ± 0.01	4.23 ± 0.01	4.24 ± 0.01
Final weight (g)	18.35 ± 0.51^c^	20.01 ± 0.42^b^	23.32 ± 0.61^a^	22.31 ± 0.17^a^
Weight gain (g)	14.11 ± 0.51^c^	15.78 ± 0.42^b^	19.08 ± 0.62^a^	18.07 ± 0.17^a^
RBWG %	332.92 ± 12.70^c^	373.25 ± 10.65^b^	450.69 ± 14.99^a^	426.21 ± 3.45^a^
SGR (%g / day)	2.61 ± 0.05^c^	2.77 ± 0.04^b^	3.04 ± 0.04^a^	2.96 ± 0.01^a^
Survival rate (%)	96.7	100	100	100
Feed intake (g feed /fish)	28.31 ± 0.28^c^	29.53 ± 0.48^b^	31.63 ± 0.37^a^	30.71 ± 0.16^a^
FCR	1.54 ± 0.04^a^	1.41 ± 0.02^b^	1.22 ± 0.03^c^	1.25 ± 0.01^c^
FER	65.00 ± 2.10^c^	70.67 ± 0.87^b^	82.09 ± 2.15^a^	79.66 ± 0.40^a^
PER	2.21 ± 0.07^c^	2.41 ± 0.03^b^	2.78 ± 0.08^a^	2.68 ± 0.07^a^
APU%	8.25 ± 0.14^c^	8.95 ± 0.17^b^	10.60 ± 0.12^a^	10.32 ± 0.18^a^
EU%	7.38 ± 0.06^b^	7.66 ± 0.09^b^	8.73 ± 0.02^a^	8.43 ± 0.18^a^

Means having the same letter in the same row are not significantly different at *P* < 0.05.

Weight gain (g) = W2 –W1.

Specific growth rate (SGR; (%g/day) = 100 (Ln W2 – Ln W1)/T.

Where, W1 and W2 are the initial and final weights, respectively, and T is the experimental period (days).

Feed conversion ratio (FCR) = feed intake/weight gain.

Protein efficiency ratio (PER) = weight gain / protein intake.

Apparent protein utilization (APU; %) = 100 [protein gain in fish (g) /protein intake in diet (g)].

Energy utilization (EU; %) = 100 [Energy gain in fish (g)/energy intake in diet (g)].

### Hematological analysis

After 24 h fasting, blood samples were collected from the caudal blood vessels of 6 fish anaesthetized with clove oil per treatment and placed into heparinized tubes. Red blood cell (RBC), total leukocyte, and platelet counts were determined using a Neubauer hemocytometer^28^. Hemoglobin (Hb) levels were measured according to^29^**,** while hematocrit (HCT) values were determined according to Ref.^30^. Mean corpuscular volume (MCV), mean corpuscular hemoglobin (MCH), and mean corpuscular hemoglobin concentration (MCHC) were calculated using the method of Ref.^31^. Differential white blood cell counts were performed following the protocol outlined by Ref.^32^.

### Serum biochemistry and immunology

For serum collection, three fish were selected from each tank, resulting in a total of nine fish per treatment. The fish were anesthetized using clove oil, and blood was drawn using a syringe without anticoagulant. After centrifugation of the blood at 3000 rpm for 15 min, the obtained serum was utilized for biochemical and immunological analyses. Total protein and albumin levels in the serum were determined colorimetrically following the methods described by Refs.^33,34^, respectively**.** The quantification of total cholesterol, as well as its various fractions (VLDL, HDL, and LDL), along with serum triglycerides, was performed using kits (Cell BioLabs, USA). Serum IgM and IgG levels were assessed using ELISA commercial kits (Cusabio, China). Complement component C3 was quantified utilizing commercial ELISA kits designed for fish Complement (MyBioSource Inc, China).

### Antioxidant enzyme activity and lipid peroxidation

Liver and intestine samples were excised separately from the dissected fish weighted, and 50 mg was immediately transferred to tubes with cold PBS (pH 7.5) which were homogenized using a tissue homogenizer. After the homogenization procedure was finished, the resulting homogenate was moved to an appropriate container for the preparation of tissue extract. Ultimately, the samples were subjected to centrifugation for a duration of 15 min at (15,000 × *g*) at 4 °C. The supernatant was kept at − 20 °C and assayed for antioxidant enzyme activity. Total superoxide dismutase (T. SOD), catalase (CAT), and glutathione peroxidase (GPx) were measured spectrophotometrically using diagnostic kits (MyBioSource Inc., China), following established methods^35–37^**.** Lipid peroxidation was determined in tissues by quantifying malondialdehyde (MDA) using the thiobarbituric acid method^38^.

### Digestive enzyme activity analysis

Segments of mid-intestine were homogenized and centrifuged. Supernatants were used to quantify digestive enzyme activity following the method of Ref.^39^. Protease activity was measured using a colorimetric assay based on hydrolysis of casein substrate and quantification of tyrosine released^40^. Lipase activity was determined by measuring the rate of hydrolysis of p-nitrophenyl palmitate to p-nitrophenol^41^. Amylase activity was assessed by the dinitrosalicylic acid (DNS) method measuring the release of maltose from starch^42^. All enzymes were analyzed using commercial reagent kits (Cusabio Biotech, China) according to the manufacturer’s protocols.

### Histopathological examination of the intestine and liver

Tissue samples from the liver and three intestinal regions (anterior, middle, posterior) were collected from 3 fish per tank and fixed in 10% neutral buffered formalin. Samples were processed for paraffin embedding and sectioned at 5 μm thickness using standard histological techniques^43^. Sections were stained with hematoxylin and eosin (H&E) and examined by light microscopy. Digital images were captured and analyzed using ImageJ software. Quantitative measurements of intestinal morphology included mucosal fold number, villus height and width, and the thickness of muscles of the intestine. All parameters were measured in triplicate on images at 20X magnification. Liver sections were evaluated for the presence of pathological changes.

### Statistical analysis

Data were tested for normality (Shapiro–Wilk test) and homogeneity of variance (Levene’s test) at a significance level of 5% prior to further analysis. One-way analysis of variance (ANOVA) was performed, followed by Duncan’s multiple range test to compare mean values between treatments at (*P* < 0.05) using SPSS (Standard Version 22.0, SPSS Inc., Chicago, Illinois). All results are presented as mean ± standard error (SE).

### Ethics approval

All the experiments were carried out in accordance with “Guidelines for the Use of Fishes in Research” published by American Fisheries Society (2014). The experimental design was approved by the Central Laboratory for Aquaculture Research (CLAR), Abassa, Egypt (Approval no. 43429), and all procedures adhered to the regulations for the care and use of fish.

## Results

### Growth performance and feed utilization

Dietary supplementation with Hilyses significantly improved growth performance and feed utilization parameters in tilapia compared to the control diet without Hilyses (Table 2). Final body weight (FBW), weight gain (WG), specific growth rate (SGR) and relative body weight gain (RBWG) all showed a significant incremental increase (*P* < 0.05) with increasing dietary levels of Hilyses up to 2 g/kg diet. No significant differences in survival rate were observed among the dietary treatments, although numerically survival was higher in the Hilyses groups compared to control. Feed efficiency ratio (FER), protein efficiency ratio (PER), apparent protein utilization (APU), and energy utilization (EU) showed significant improvements with Hilyses supplementation, with the highest values observed in the 2 g/kg group (P < 0.05). There was a significant decrease (*P* < 0.05) in feed conversion ratio (FCR) observed with increasing dietary levels of Hilyses.

### Proximate composition

The proximate composition analysis of the fish carcasses showed no significant differences in ash contents between the dietary treatments (Table 3). However, crude protein content showed a significant incremental increase (P < 0.05) with higher dietary Hilyses. Fish fed the control diet without Hilyses had the lowest carcass crude protein content. Moisture contents were significantly (*P* < 0.05) high in Hilyses fed groups. An opposite trend was noticed for crude lipid content, which decreased significantly with the highest dietary Hilyses; 3 g/kg.

**Table 3:** Whole body composition (%, of fresh weight basis (g)) of Nile tilapia as affected by dietary additives of Hilyses® for 8 weeks.

	Hilyses^®^ (g/ kg)			
	0.0	1	2	3
Moisture	71.46 ± 0.03^b^	72.29 ± 0.06^a^	72.40 ± 0.21^a^	72.53 ± 0.05^a^
Crude protein	16.10 ± 0.02^b^	16.82 ± 0.23^b^	17.84 ± 0.34^a^	17..90 ± 0.16^a^
Total lipids	7.03 ± 0.09^a^	6.67 ± 0.30^ab^	6.49 ± 0.01^ab^	6.31 ± 0.04^b^
Ash	3.47 ± 0.03	3.64 ± 0.04	3.66 ± 0.17	3.69 ± 0.23

Means having the same letter in the same row are not significantly different at *P* < 0.05.

### Hematological parameters

The effects of dietary Hilyses supplementation on hematological parameters are presented in Table 4. The percentage counts of all three differential leukocytes—neutrophils, lymphocytes, and monocytes—showed significant stepwise increases with higher dietary Hilyses, with the highest values observed in the 2 g/kg group (*P* < 0.05). Total erythrocyte (RBC) count, total leukocyte count, hemoglobin, hematocrit, and mean corpuscular volume (MCV) also showed similar trends of significant incremental increases with higher dietary Hilyses up to an optimum at 2 g/kg diet (*P* < 0.05).

**Table 4:** Hematological parameters of Nile tilapia as affected by dietary additives of Hilyses^®^ for 8 weeks.

Items	Hilyses^®^ (g/kg)			
	0.0	1	2	3
Leukogram				
Neutrophil (%)	9.61 ± 1.02^b^	10.66 ± 0.23^ab^	12.86 ± 0.62^a^	12.00 ± 0.81^ab^
Lymphocytes (%)	84.00 ± 0.81^b^	84.33 ± 0.23^b^	87.08 ± 1.47^a^	85.00 ± 0.40^ab^
Monocytes (%)	1.66 ± 0.23^c^	2.00 ± 0.10^bc^	2.33 ± 0.23^a^	2.33 ± 0.24^a^
Eosinophils (%)	1.33 ± 0.47	1.66 ± 0.23	1.35 ± 0.10	1.41 ± 0.11
Erythrogram				
RBCs (× 10^12^ /L)	2.07 ± 0.08^c^	2.47 ± 0.07^b^	3.10 ± 0.04^a^	2.84 ± 0.05^a^
Hb (g/dl)	6.00 ± 0.22^c^	7.16 ± 0.53^b^	8.96 ± 0.31^a^	8.26 ± 0.16^a^
HCT (%)	17.20 ± 0.12^c^	20.83 ± 0.56^b^	25.93 ± 0.49^a^	21.02 ± 0.51^b^
MCV (fl)	82.60 ± 0.25^c^	83.59 ± 10.19^b^	84.30 ± 0.24^a^	83.70 ± 0.08^ab^
MCH (dl)	28.16 ± 0.14	28.86 ± 0.14	28.89 ± 0.07	28.88 ± 0.07
MCHC (g/dl)	34.60 ± 0.72	34.60 ± 0.85	34.60 ± 1.31	34.60 ± 0.91
Total leukocyte count (× 10^9^ /L)	0.90 ± 0.08^c^	1.06 ± 0.12^bc^	2.26 ± 0.24^a^	1.43 ± 0.15^b^

Data were presented as mean ± standard error. Means having the same letter in the same row are not significantly different at *P* < 0.05.

*RBCs* Red blood cells, *HCT* Hematocrit, *Hb* Hemoglobin, *MCV* mean corpuscular volume, *MCHC* Mean corpuscular hemoglobin concentration.

### Serum biochemistry and immune function

Fish fed diets containing 2 and 3 g kg^−1^ Hilyses exhibited significantly higher levels of serum total protein (TP), albumin, C3, IgM, and IgG compared to the other treatment diets (*P* < 0.05; Table 5). Additionally, diets supplemented with 3 g kg-1 Hilyses showed significantly reduced total cholesterol and triglyceride levels compared to the control diets (*P* < 0.05). Serum HDL levels were highest in fish fed diets supplemented with 2 and 3 g kg^−1^ Hilyses, while LDL and VLDL values were lowest in fish fed Hilyses- supplemented diets compared to the control diets.

**Table 5:** Immunological and blood chemistry parameters of Nile tilapia as affected by dietary additives of Hilyses® for 8 weeks.

Items	Hilyses® (g/ kg)			
	0.0	1	2	3
Total protein (g/dl)	6.75 ± 0.20^c^	8.40 ± 0.11^b^	14.30 ± 0.86^a^	13.90 ± 0.40^a^
Albumin (g/dl)	5.78 ± 0.10^c^	6.62 ± 0.12^b^	12.70 ± 0.63^a^	12.10 ± 0.98^a^
C3(µg/ml)	356.00 ± 8.08^b^	416.00 ± 38.10^b^	597.00 ± 21.36^a^	515.00 ± 40.99^a^
IgM (µg/ml)	15.91 ± 1.43^c^	26.06 ± 1.22^b^	39.37 ± 4.56^a^	40.13 ± 1.32^a^
IgG (µg/ml)	39.21 ± 2.58^c^	54.90 ± 2.02^b^	72.41 ± 5.37^a^	72.16 ± 1.35^a^
Cholesterol (mg/dl)	439.00 ± 25.98^a^	359.00 ± 23.67^b^	329.00 ± 20.20^bc^	266.50 ± 17.21^c^
Triglyceride(mg/dl)	742.50 ± 33.19^a^	667.00 ± 24.82^ab^	591.00 ± 22.51^b^	463.00 ± 25.98^c^
HDL(mg/dl)	167.00 ± 8.66^b^	202.00 ± 6.92^b^	316.00 ± 20.78^a^	330.00 ± 17.32^a^
LDL(mg/dl)	166.90 ± 7.79^a^	77.30 ± 5.71^b^	57.50 ± 5.13^bc^	47.30 ± 6.52^c^
VLDL(mg/dl)	120.80 ± 10.50^a^	83.40 ± 9.11^b^	64.40 ± 8.08^b^	58.97 ± 6.16^b^

*C3* complement 3, *IgM* immunoglobulin M, *IgG* Immunoglobulin G, *HDL* high-density lipoprotein, *LDL* low-density lipoprotein, *VLDL* very low-density lipoprotein.

Means having the same letter in the same row are not significantly different at *P* < 0.05.

### Antioxidant enzymes and lipid peroxidation

The effects of dietary Hilyses on antioxidant enzyme activities in the liver and intestine are shown in Table 6. Activities of catalase (CAT), glutathione peroxidase (GPX) and total superoxide dismutase (T.SOD), representing major cellular antioxidant defense mechanisms, were significantly higher in the 2 g/kg Hilyses group compared to the control. Concurrently, the MDA levels, as an index of lipid peroxidation, were significantly lower in the 2 g/kg Hilyses group (P < 0.05), indicating reduced oxidative damage to lipids. These results suggest dietary Hilyses enhanced antioxidant status and alleviated oxidative stress in tilapia, with optimal effects at 2 g/kg supplementation.

**Table 6:** Antioxidant enzymes assay and lipid peroxidation of Nile tilapia liver and intestine as affected by dietary additives of Hilyses^®^ for 8 weeks.

Items	Hilyses^®^ (g/ kg)			
	0.0	1	2	3
Intestine				
MDA (nmol/mg protein)	64.35 ± 0.50^a^	54.64 ± 0.63^b^	40.67 ± 1.08^c^	41.51 ± 0.95^c^
CAT (U/mg protein)	164.77 ± 9.04^d^	196.22 ± 3.31^c^	363.95 ± 8.07^a^	261.31 ± 7.56^b^
GP_X_ (U/mg protein)	392.56 ± 2.78^d^	411.60 ± 4.43^c^	502.26 ± 2.18^a^	442.33 ± 3.14^b^
T-SOD (U/mg protein)	667.39 ± 2.93^d^	701.14 ± 1.44^c^	918.36 ± 1.143^a^	859.71 ± 1.29^b^
Liver				
MDA (nmol/mg protein)	73.81 ± 1.01^a^	64.24 ± 0.89^b^	52.35 ± 0.83^c^	53.64 ± 0.74^c^
CAT (U/mg protein)	111.63 ± 0.72^d^	274.83 ± 0.91^c^	374.66 ± 1.12^a^	304.85 ± 0.87^b^
GP_X_ (U/mg protein)	370.50 ± 0.78^d^	416.99 ± 0.76^c^	550.12 ± 0.72^a^	455.70 ± 0.77^b^
T-SOD (U/mg protein)	789.29 ± 0.85^d^	815.47 ± 0.79^c^	942.31 ± 0.82^a^	846.61 ± 0.48^b^

*MDA* malondialdehyde, *CAT* catalase, *GPx* glutathione peroxidase, *T-SOD* total superoxide dismutase.

Means having the same letter in the same row are not significantly different at *P* < 0.05.

### Digestive enzyme activity

The effects of dietary Hilyses on digestive enzyme activity in the tilapia intestine are presented in Table 7. Significant numerical increases in protease and lipase activities were observed with Hilyses supplementation (P < 0.05). Protease activity peaked at 2 g/kg Hilyses, while lipase activity was maximized at both 2 and 3 g/kg supplementation levels. In contrast, amylase activity was lower in the Hilyses groups compared to the control. The results indicate potential beneficial effects of Hilyses on protein and lipid digestion in tilapia.

**Table 7:** Digestive enzymes of Nile tilapia as affected by dietary additives of Hilyses^®^ for 8 weeks.

	Hilyses^®^ (g/ kg)			
	0.0	1	2	3
Protease (U/L)	2301 ± 52.53^c^	2566 ± 43.22^b^	2795 ± 20.20^a^	2641 ± 29.44^ab^
Lipase (U/L)	1381 ± 16.74^c^	1566 ± 24.82^b^	1746 ± 22.15^a^	1817 ± 16.74^a^
Amylase (U/L)	1790 ± 10.96^a^	1668 ± 12.86^b^	1503 ± 9.81^c^	1520 ± 11.32^c^

Means having the same letter i n the same row are not significantly different at *P* < 0.05.

### Intestinal and hepatic histopathology

**Figure 1:**
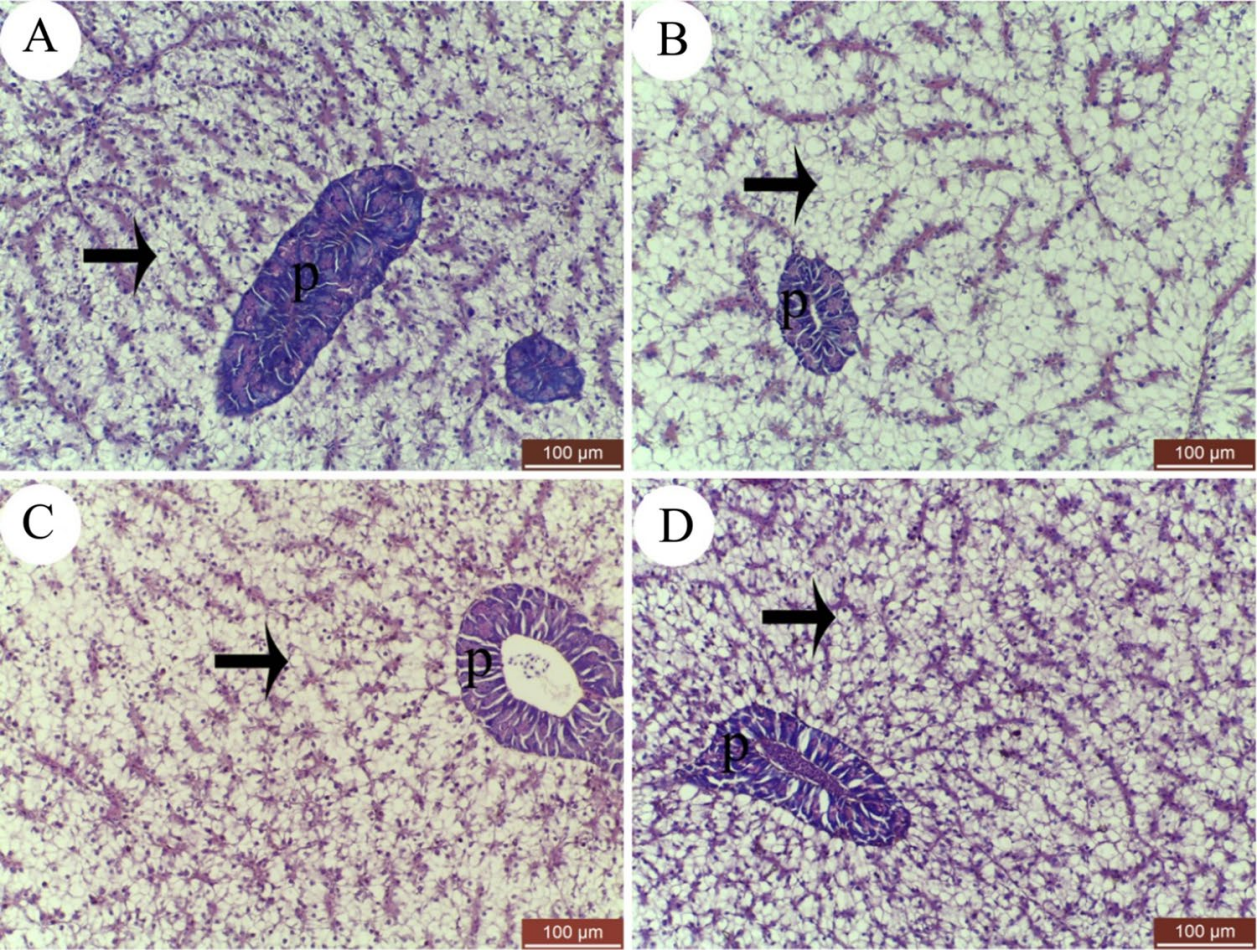
Hematoxylin and eosin (H&E) staining of Nile tilapia (*O. niloticus*) hepatopancreas fed diets with varying Hilyses® levels. (**A**) Control group, (**B**) 1 g kg^−1^ diet, (**C**) 2 g kg^−1^ diet, (**D**) 3 g kg^−1^ diet. Hepatocytes (black arrows) and pancreatic tissue (P) are indicated.

The liver of Nile tilapia*, O. niloticus,* showed typical histological structure. It consists of polygonal hepatocytes arranged in the form of hepatic cords. The pancreatic tissues were observed in between hepatic cords (Fig. 1A–D). The cytoplasm of hepatocytes was acidophilic in control group (0.0 g kg^−1^ Hilyses diet) (Fig. 1A). The hepatocytes׳ cytoplasm of the group fed with 1 g kg^−1^ Hilyses diet was lighter than control group which indicates increase in lipids deposition inside hepatocytes (Fig. 1B). Little cytoplasmic acidophilia was noticed in the groups fed with 2 and 3 g kg^−1^ Hilyses diet (Fig. 1C and D respectively). This result indicates that lipids deposition was lower at 2 and 3 g kg^−1^ Hilyses diet than at 1 g kg^−1^ Hilyses diet and control.

**Figure 2:**
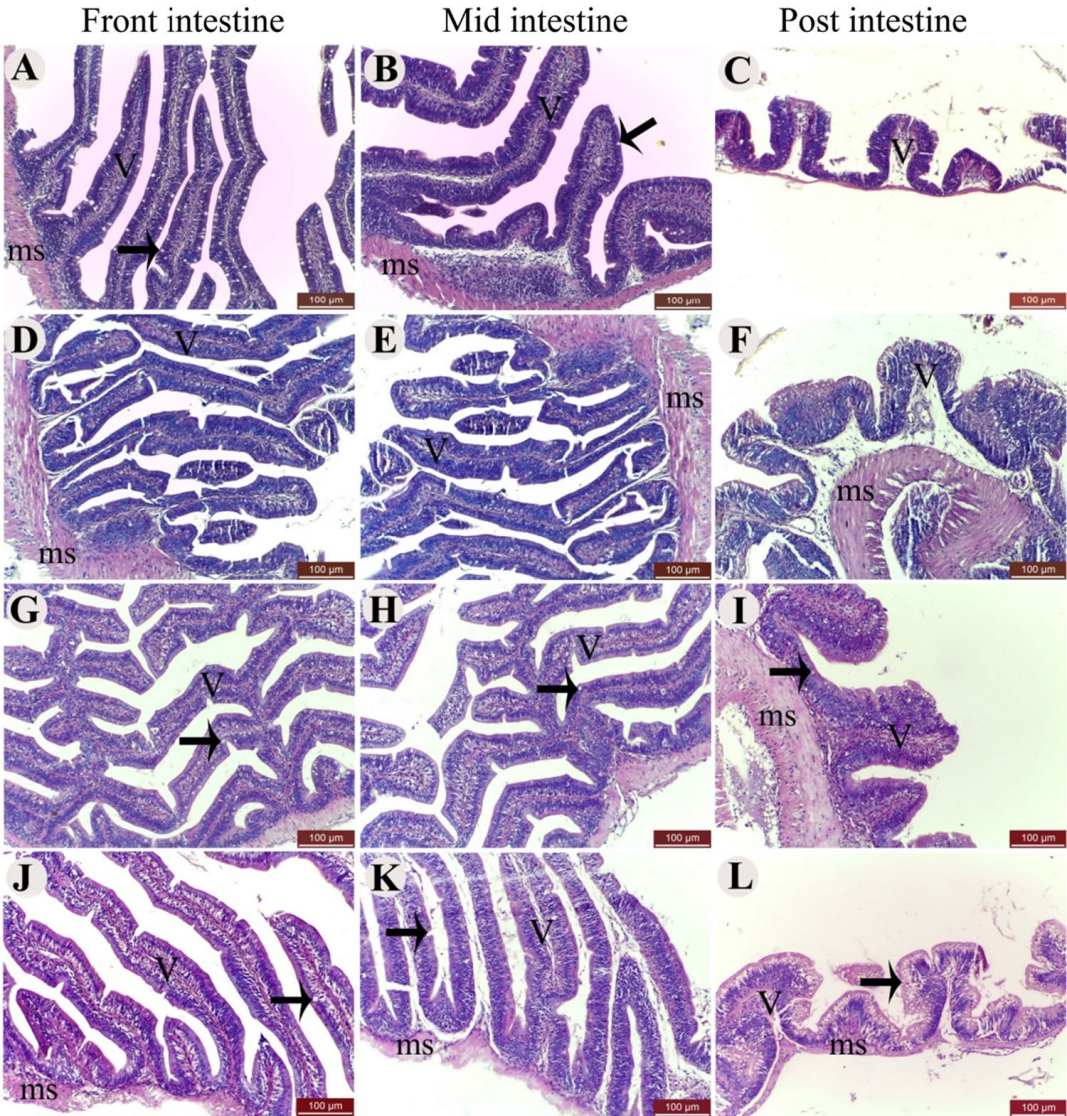
The H&E staining of front, middle, and posterior intestine of Nile tilapia (*O. niloticus*) fed diets supplemented with varying Hilyses® levels. Control (**A**–**C**), 1 g kg^−1^ diet (**D**–**F**), 2 g kg^−1^ diet (**G**–**I**), and 3 g kg^−1^ diet (**J**–**L**). Intestinal villi (V), goblet cells (arrows), and tunica muscularis smooth muscle (ms) are indicated.

The intestinal histological structure of *O. niloticus* appeared normal. The front and middle intestine were lined with a simple columnar epithelium containing goblet cells. The tunica submucosa serves as a barrier between the villi and the tunica muscularis. The tunica muscularis is composed of inner circular and outer longitudinal smooth muscles and is covered by the mesothelium of the tunica serosa. The posterior intestine is characterized by short mucosal folds (Fig. 2A–L). There was characteristic branching of the villi in the group fed with 2 g kg^−1^ Hilyses diet in comparison to other groups (Fig. 2G,H).

**Figure 3:**
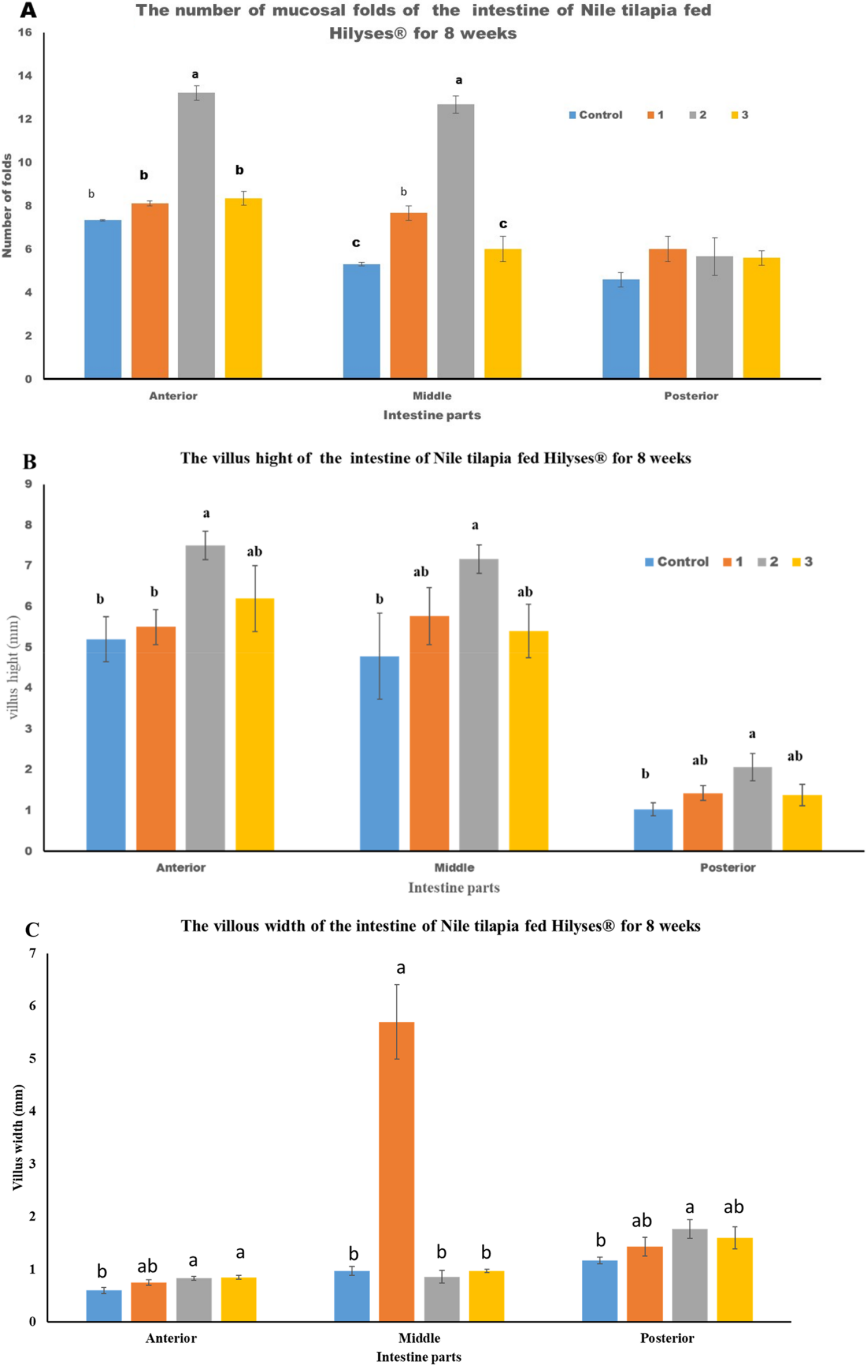

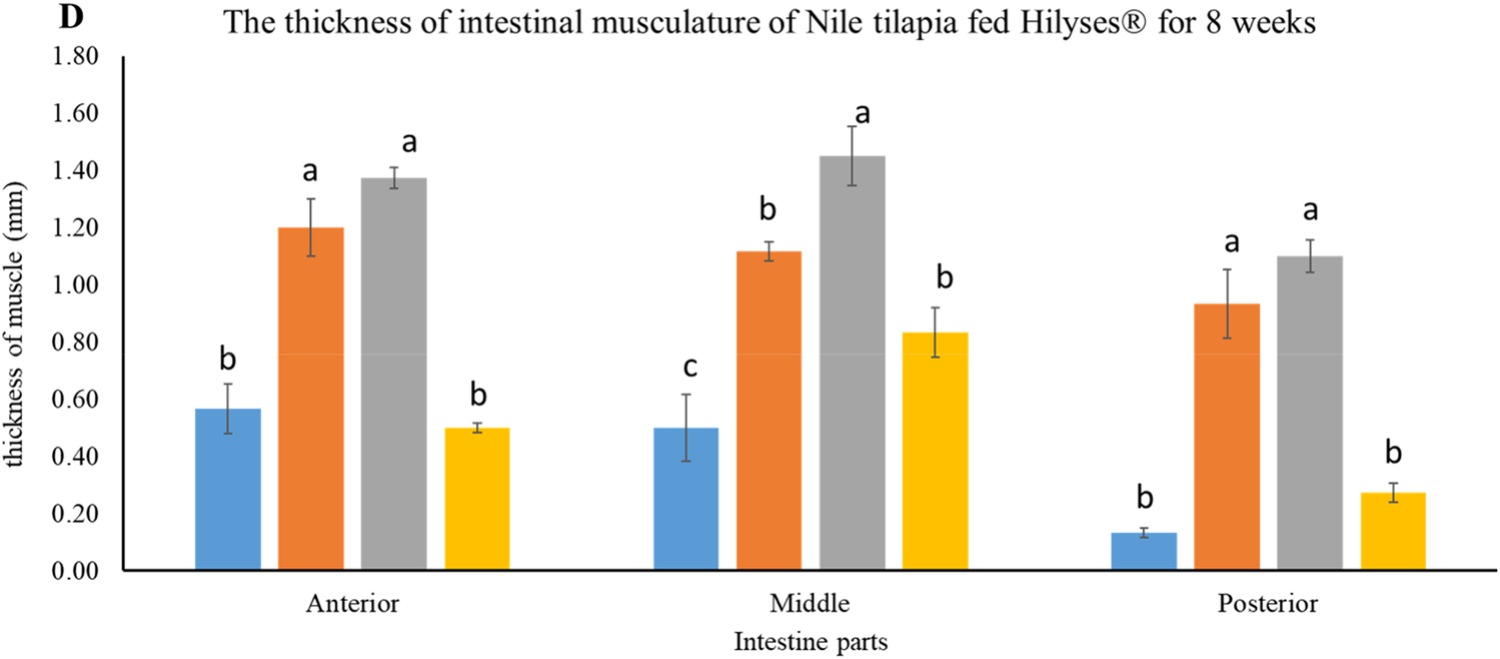
Effect of diets supplemented with varying levels of Hilyses® on intestinal morphology of Nile tilapia (*O. niloticus*). (**A**) Number of mucosal folds, (**B**) villous height, (**C**) villous width, and (**D**) thickness of intestinal musculature. Data are presented as mean ± SEM (n = 3). Bars with the same color with different letters are significantly different at P < 0.05, using Duncan's post hoc test.

Concerning the effect of Hilyses on the intestine morphometric, it was observed that the number of villi, its thickness, and width at the different parts of the intestine were significantly affected by the two high concentrations (2 and 3 g kg^−1^) compared to the control (Fig. 3A–C). Also, the intestinal muscle thickness showed an obvious increase in the groups fed Hilyses at different concentrations (Fig. 3D).

## Discussion

The use of feed additives to enhance diverse aspects of fish health and performance has become increasingly prevalent in aquaculture. This has sparked intense competition between feed additive manufacturers striving to formulate proprietary functional ingredient blends that provide comprehensive benefits in a single product. The current study demonstrated that dietary supplementation with Hilyses, a fermented *S. cerevisiae* product, led to significant improvements in feed utilization efficiency and growth performance metrics like weight gain, specific growth rate, and relative body weight gain in Nile tilapia at supplementation levels of 2.0 and 3.0 g/kg diet. There were no significant differences in survival rate among the dietary treatment groups, although numerically survival was slightly higher in the groups receiving Hilyses supplementation compared to the unsupplemented control group. The feed conversion ratio showed no significant treatment differences either. However other feed utilization parameters like feed efficiency ratio, protein efficiency ratio, apparent protein utilization, and energy utilization exhibited significant stepwise improvements with increasing dietary Hilyses up to an optimal supplementation level of 2.0 g/kg.

Hilyses is a commercial nutraceutical product containing a proprietary blend of β-glucans, free nucleotides, glutamine, peptides, mannan oligosaccharides, and other bioactive compounds derived from the fermentation of *S. cerevisiae* yeast. The various constituents in this mixture could elicit beneficial effects on growth performance through several potential mechanisms of action as prebiotics or immunostimulants, either working individually or in synergistic combinations to promote digestion, enhance digestive enzyme activity, favorably alter the gastrointestinal microbiota, and modify the intestinal mucosal environment in ways that may improve feed utilization efficiency, nutrient absorption, and subsequently growth performance^44,45^. For example, the β-glucans comprise 60–70% of the polysaccharides making up the cell wall of *S. cerevisiae* yeast and have been extensively studied for their growth-promoting effects when included in fish feeds^46–48^. The inclusion of nucleotides in the Hilyses formulation has also been shown to significantly improve overall performance metrics like growth and feed efficiency across different fish species^49,50^. Additionally, incorporating nucleotide supplements into fish feeds with reduced fishmeal content as alternative protein sources has been found to enhance the effectiveness of the feeds, resulting in improved growth, health, and reduced dependence on scarce fishmeal resources^51^.

The enhancements in growth performance parameters observed in this study with Hilyses supplementation may potentially be attributed in part to the inclusion of glutamine as one of the active components in the product. Glutamine is an essential functional amino acid that plays crucial regulatory roles in fish nutrition and physiology. Numerous studies have thoroughly documented the involvement of glutamine in growth regulation, appetite stimulation, protein synthesis, and skeletal muscle growth^52–55^. The application of Hilyses as a dietary feed supplement has been investigated in several previous studies utilizing a range of inclusion levels and feeding durations. For example, significant improvements in the growth performance of Nile tilapia juveniles were reported when provided diets supplemented with 2% and 4% Hilyses for a period of two months^19^. Similar positive results on growth parameters were observed in earlier studies^7,22^, where rainbow trout exhibited enhanced growth rates when fed diets containing Hilyses. Therefore, the growth performance improvements noted in the current investigation align closely with the benefits reported in previous literature when supplementing fish feeds with Hilyses.

In the present study, supplementing diets with increasing levels of Hilyses had no significant effects on the moisture or ash contents of tilapia carcasses. However, crude protein content showed a significant stepwise increase concurrent with higher dietary inclusion levels of Hilyses. An opposite trend was noticed for crude lipid content, which decreased significantly as more Hilyses was incorporated into the diets. These results are consistent with previous findings^56,57^ who reported that supplementing diets with live *S. cerevisiae* yeast resulted in increased protein deposition and reduced lipid levels in tissue composition analyses for either Nile or Galilee tilapia *Sarotherodon galilaeus*. However, contradictory to the present results, no significant alterations in whole-body composition parameters were reported when rainbow trout were fed diets enriched with Hilyses as the supplement^7,21^. Therefore, the effects of yeast-based functional feed additives on body composition appear to be somewhat inconsistent between different studies and fish species.

The fermentation process used to produce Hilyses likely plays an important role in enhancing its bioactivity and efficacy as a feed additive. The fermentation of *S. cerevisiae* yeast using specialized proprietary processes results in the release and concentration of various bioactive compounds, including cell wall components like β-glucans, nucleotides, peptides, and other metabolites. These fermentation-derived constituents have been shown to exhibit superior bioavailability and potency compared to non-fermented yeast preparations^57^. The synergistic interactions between these diverse bioactive compounds present in the Hilyses formulation may underlie the comprehensive improvements observed across the different physiological parameters in the current study.

Hematological blood parameters serve as very informative health indicators in fish, providing insights into the nutritional condition, physiological stress levels, immune function, and effects of environmental factors^58^. In the current study, dietary Hilyses supplementation induced significant improvements in total blood cell counts, including higher numbers of all three major differential leukocyte types—neutrophils, lymphocytes, and monocytes. Total erythrocyte (red blood cell) count, total leukocyte count, hemoglobin, hematocrit, and mean corpuscular volume also showed significant stepwise increases with higher inclusion of Hilyses up to an optimal level of 2.0 g/kg diet. These results are consistent with the hematology improvements reported in previous studies supplementing diets of Nile tilapia with S*. cerevisiae* yeast additives, including the findings of^19,59^. The hematological benefits of yeast additives are thought to be related to stimulatory effects on hematopoiesis induced by active components like β-glucans and MOS present in the yeast cell wall matrix^60^. Specifically, β-glucans have proven immunostimulatory properties associated with enhanced innate immune responses and increased leukocyte production and mobilization^61^, which likely contributed to the observed blood profile improvements. Serum biochemistry parameters like protein levels, enzymes, and electrolytes provide valuable insights into health conditions in animals^62^. They indicate the functional status of internal organs, nutritional condition, and metabolic state^63^. In this study, supplementing diets with Hilyses led to increased total serum protein levels. This suggests Hilyses supported fish immunity during physiological stress. The immune response of Nile tilapia may be enhanced through optimal dietary active immunostimulatory polysaccharide supplementation, as the elevated proteins align with previous tilapia studies^64^. Similarly, the yeast derivative β-glucan was found to increase total serum proteins in Nile tilapia^65^. The addition of Hilyses to diets also substantially improved immunological markers including complement component C3, immunoglobulin M (IgM), and IgG. Comparable immunological improvements from yeast-derived β-glucans were reported in Nile tilapia^66,67^. This further indicates the immunostimulatory properties of Hilyses components like β-glucans.

The serum levels of high-density lipoprotein (HDL), low-density lipoprotein (LDL), and triglycerides (TG) are important indicators that represent the status of lipid metabolism^68^. In this study, the addition of Hilyses to the diet resulted in a substantial decrease (p < 0.05) in LDL, total cholesterol, and triglyceride levels compared to the other diets. Conversely, the fish that were given a diet containing 2 and 3 g/kg of Hilyses showed the highest levels of HDL. In contrast, the lowest levels of LDL were found in the fish that were given a diet containing 3 g/kg of Hilyses. There were no statistically significant variations (P > 0.05) in VLDL levels between the groups supplemented with Hilyses and the control diet. This finding is consistent with the results reported by^68^. The improved blood lipid profile in fish fed Hilyses may be attributed to the improved nutrient assimilation and lipid metabolism.

In this study, 2 g/kg Hilyses significantly increased intestinal and hepatic CAT, GPX, SOD activity while decreasing MDA compared to control diets. This suggests dietary Hilyses enhances antioxidants and reduces oxidative damage, likely due to an increased influx of antioxidant stimulators^69^. Similar antioxidant improvements from immunostimulant mixtures occurred in tilapia^64^ and grass carp^70^.

The growth and performance of fish is closely associated with the functioning of digestive enzymes. Aquatic species primarily utilize enzymes released from the liver and pancreas to carry out digestion, which significantly impacts the absorption of nutrients^70^. In this study, dietary Hilyses decreased amylase activity but increased protease and lipase to varying degrees. The improvements in protease and lipase activity may enhance protein and lipid digestion, allowing for increased nutrient absorption and promoting growth. These results agree with previous studies showing immunostimulant mixtures can beneficially affect digestive enzymes in fish^71^. Specifically, glutamine has been shown to stimulate production of digestive enzymes and regulate absorption of amino acids^72^. Similar effects of yeast supplements on enzyme activity were seen in shrimp fed fermented soybean meal^73^.

The overall health of fish correlates closely with the integrity of the intestinal tract and its absorptive capabilities^74^. Here, Hilyses significantly improved intestinal morphology as indicated by increased villus height and width, mucosal fold number, and muscular thickness. These enhancements likely improved the general performance of tilapia by facilitating greater nutrient absorption. Comparable beneficial effects on intestine structure were reported in tilapia fed yeast extracts^75^ and carp fed yeast hydrolysates^76^. Fermented yeast also increased villi length and goblet cell number in tilapia^77^. These intestinal benefits may be attributed to mannan oligosaccharides in yeast inhibiting pathogenic bacteria attachment to the intestinal lining, preventing inflammation and damage to the villi and microvilli^78^.

Overall, the findings of this study indicate that dietary supplementation with the fermented *S. cerevisiae* product Hilyses can effectively enhance the growth performance, feed utilization, nutrient composition, hematological and immunological parameters, antioxidant status, and digestive function of juvenile Nile tilapia. The unique blend of bioactive compounds present in Hilyses, derived through the fermentation process, appear to be responsible for the comprehensive beneficial effects observed across multiple physiological indices. The optimal supplementation level was determined to be 2.0 g/kg diet, which provided the most pronounced improvements. These results highlight the potential of Hilyses as a valuable functional feed additive to improve the productivity and health of Nile tilapia in aquaculture systems.

## Data Availability

All data that support the findings of this study are available upon request from the corresponding author.
